# Correlation between postmortem microbial signatures and substance abuse disorders

**DOI:** 10.1371/journal.pone.0274401

**Published:** 2022-09-26

**Authors:** Gulnaz T. Javan, Tiara Wells, Jamese Allen, Silvia Visona, Matteo Moretti, Craig Tipton, Latia Scott, Sheree J. Finley

**Affiliations:** 1 Department of Physical Sciences and Forensic Science Programs, Alabama State University, Montgomery, Alabama, United States of America; 2 Department of Public Health, Experimental and Forensic Medicine, University of Pavia, Pavia, Italy; 3 RTL Genomics, Lubbock, Texas, United States of America; 4 Department of Biological Sciences, Texas Tech University, Lubbock, Texas, United States of America; 5 Department of Biological Sciences, Delaware State University, Dover, Delaware, United States of America; 6 College of Agriculture, Virginia State University, Petersburg, Virginia, United States of America; University of Minnesota Twin Cities, UNITED STATES

## Abstract

The microbiota gut-brain-axis is a bidirectional circuit that links the neural, endocrine, and immunological systems with gut microbial communities. The gut microbiome plays significant roles in human mind and behavior, specifically pain perception, learning capacity, memory, and temperament. Studies have shown that disruptions in the gut microbiota have been associated with substance use disorders. The interplay of gut microbiota in substance abuse disorders has not been elucidated; however, postmortem microbiome profiles may produce promising avenues for future forensic investigations. The goal of the current study was to determine gut microbiome composition in substance abuse disorder cases using transverse colon tissues of 21 drug overdose versus 19 non-overdose-related cases. We hypothesized that postmortem samples of the same cause of death will reveal similar microbial taxonomic relationships. We compared microbial diversity profiles using amplicon-based sequencing of the 16S rRNA gene V4 hypervariable region. The results demonstrated that the microbial abundance in younger-aged cases were found to have significantly more operational taxonomic units than older cases. Using weighted UniFrac analysis, the influence of substances in overdose cases was found to be a significant factor in determining microbiome similarity. The results also revealed that samples of the same cause of death cluster together, showing a high degree of similarity between samples and a low degree of similarity among samples of different causes of death. In conclusion, our examination of human transverse colon microflora in decomposing remains extends emerging literature on postmortem microbial communities, which will ultimately contribute to advanced knowledge of human putrefaction.

## Introduction

The bidirectional interplay of the brain and the gut microbiota, termed microbiota-gut-brain (MGB) axis, participates in a significant role in brain function, mood, and behavior. Gut microbiota receives its first inoculation of microbes during birth [1], and subsequently the structure is altered via environmental elements, infections, stress, the use of drugs [2], and even after death [3]. The gut microbiota affects the host central nervous system (CNS) functions and conversely the CNS influences the microbiota composition [4–6]. Currently, there is a paucity of information on how gut microbes respond to the influence of commonly abused drugs such as opioids and cocaine. Previous studies suggest that commonly abused drugs dysregulate the gut microbiota, which is crucial given the effects of gut microbes in modulating addictive behaviors associated with drug abuse [4, 5, 7, 8]. However, it is not known which drug type (e.g., heroin, cocaine) or MGB body sites host the most accurate succession of microbes after death. Identifying the postmortem microbial diversity of tissues in the gut region affected by drug abuse has potential to establish the rate at which microorganisms predominate.

The MGB axis has been associated with gastrointestinal disease (e.g., leaky gut), CNS diseases as well as substance use disorders (SUDs) [9]. As a result, the MGB axis has become a target for therapeutics and a possible cause of drug side effects [10, 11]. More recently, Xu et al. (2017) have found a distinguished intestinal microbiota profile among living individuals with SUDs using their fecal samples. However, human tissue samples are more challenging to obtain, but fecal specimens do not provide insight into the differences in the gut microbiota among individuals who have died due to drug overdose. Furthermore, the retrieval of human DNA from fecal matter is difficult due to the presence of bile salts and plant polysaccharides [12]. Another major limitation is that some gut microbes may be present at levels lower than the limit of detection in fecal samples [13]. Therefore, some microbial taxa are not detected in feces.

Gut microorganisms associate with humans to form endosymbiotic networks of intergenomic associations. For example, Firmicutes in the gut have been shown to ferment carbohydrates into an array of short-chain fatty acids (SCFAs) and decreased intestinal barrier function has been shown to be attributable to a deficiency of SCFAs [14, 15]. As a result, intestinal pathogens and their metabolites cross barriers in the gut and activate immune responses, which form a “leaky gut” that affects the development of major depressive disorders [16].

The human gut microbiota is primarily dominated by two bacterial phyla, Bacteroidetes and Firmicutes which characterize more than 90% of the total microbial community [17]. Studies have shown that morphine significantly alters gut microbiota, triggering preferential proliferation of gram-positive Firmicutes and reduction of gram-negative Bacteroidetes [18], consistent with microbiota associations with drug addiction. The coexistence of these two phyla in the gut indicates diminished competition for available nutrition through cooperation or specialization [19]. As drug overdose deaths continue to increase, it is particularly challenging for medical examiners due to the limitations of current toxicology techniques as they were not designed to identify the most challenging opioids (e.g., fentanyl) [20]. Furthermore, common equipment used in forensic labs are not able to detect small amounts of drugs in the victim’s system, even if the amount is proven to be deadly [21]. Therefore, an alternate or supplemental technique is needed to assist in determining drug overdose as the cause of death. Generally, the determination of the time of death, also known as the postmortem interval (PMI), can be problematic depending on extrinsic factors such as temperature. Identifying a unique gut microbiota profile at known PMIs may provide additional use in the field of forensics.

The goal of the current study was to determine transverse colon microbiome composition in cases that succumbed to SUDs in 40 cadavers: 21 drug overdose cases versus 19 natural deaths. We hypothesized that tissues obtained from cases that died from drug overdose will demonstrate microbial taxonomic categories containing similar phyla. To test this hypothesis, we compared microbial community profiles using amplicon-based sequencing of the V4 hypervariable region of the 16S rRNA gene.

## Materials and methods

### Cadaver colon sample collection

COVID-19-infected specimens were excluded from this study. Postmortem samples included 34 male and six female corpses obtained from the Department of Public Health in Experimental and Forensic Medicine at University of Pavia in Pavia, Italy. Out of the four sections of the colon—ascending, transverse, descending, and sigmoid colon, we only collected transverse colon samples. We chose this section since studies have shown that *Clostridium* is present in this location of the colon antemortem [22] and will exhibit the “Postmortem Clostridium Effect” (PCE), which refers to facultative anaerobic *Clostridium* spp. that are ubiquitous during human decomposition [23]. Demographic data were collected on each of the 40 corpses (i.e., ethnicity, height, weight, PMI, cause of death, manner of death, SUDs status, and drug substance) (S1 Table in S1 File). The study was approved prospectively by the Alabama State University Institutional Review Board (IRB) number 2019098. In case of deceased subjects, the consent is not required, as the samples had been taken anyway for forensic purposes and because it is not possible to contact the next of kin in such circumstances. The reference law is the authorization n9/2016 of the guarantor of privacy, then replaced by REGULATION (EU) 2016/679 OF THE EUROPEAN PARLIAMENT AND OF THE COUNCIL. Cadaver tissues were collected according to previously reported protocols. Organs were transported from University of Pavia on dry ice to the Thanatos Lab on the campus of Alabama State University, and specimens were stored at -80°C until further analyses [23].

### DNA isolation from colon samples

DNA extraction was performed using the traditional phenol-chloroform method as previously described [24]. Briefly, 2X TENS (100 mM Tris–HCl [pH 8.0], 40 mM EDTA, 200 mM NaCl, 2% SDS) buffer was added in Lysing Matrix E tubes (MP Biomedicals, Santa Ana, CA). Using a sterile surgical scalpel, 10 mg-size pieces of each sample were cut and placed in the tubes. 0.5 ml phenol:chloroform:isoamyl alcohol 25:24:1 (Sigma-Aldrich, St. Louis, MO) (TE saturated, pH 8.0) and 0.5 ml of 2X TENS buffer was added to each tube. The tubes were homogenized using a mechanical beadbeater at speed 6 for 40 s and centrifuged at 16,000 RCF for five min. The supernatants were transferred to Phase Lock Gel Heavy tubes (VWR International, Radnor, PA) containing 0.3 ml of 7.5 M ammonium acetate. An equal volume of chloroform (Sigma-Aldrich, St. Louis, MO) was added to each tube and mixed gently by inversions (10x) then centrifuged at 16,000 RCF for five minutes. 0.6 volume of ice-cold isopropanol (Sigma-Aldrich, St. Louis, MO) and then 3 μl GlycoBlue (Thermo Fisher Scientific, Waltham, MA) was added at the bottom of the tubes. The tubes were then placed into a –80°C freezer then centrifuged at 16,000 RCF for five min. Following centrifugation, the isopropanol was decanted, and the pellets were washed with cold 80% ethanol and allowed to dry. The pellets were resuspended by adding 100 μl final volume TE buffer.

### Illumina MiSeq sequencing

DNA extracts were analyzed for library prep and 16S rRNA gene sequencing on the Illumina MiSeq platform, as previously described [25, 26]. Briefly, the V4 hypervariable region of the 16S rRNA gene was amplified for sequencing with primers 515F-806R (Forward: 5’GTG CCA GCM GCC GCG GTA A-3’, Reverse: 5’-GGA CTA CHV GGG TWT CTA AT-3’) [25]. Primers for the first step were constructed using the fragment-specific forward and reverse primers (515F-806R) with the Illumina i5 and i7 sequencing primers added to the 5′-end of each, respectively. Amplification products were visualized with eGels (Life Technologies, Carlsbad, CA). Products were then pooled equimolar, and each pool was size selected in two rounds using SPRI select beads (BeckmanCoulter, Brea, CA) in a 0.7 ratio for both rounds. Size selected pools were then quantified using Qubit 2.0 fluorometer (Life Technologies, Carlsbad, CA) and loaded on an Illumina MiSeq 2x300 flow cell at 10 pM.

### Bioinformatic processing

The raw sequence data were analyzed using a standard microbial diversity analysis pipeline which includes paired read merger, denoising, chimera checking, OTU clustering and identification at 97% sequence similarity, as previously reported [25, 27]. Paired reads were merged using PEAR [28]. The UCHIME chimera detection software was used to distinguish chimeras in de novo mode [29]. Further, to eliminate noise from within each sequence, remaining sequences were then corrected base-by-base. Each sample read was clustered into OTUs (at 97% identity) using the UPARSE algorithm [30]. Next, to determine taxonomic classifications, the reads were globally aligned for OTU selection using the USEARCH global algorithm [31] using a database of high-quality, 16S rRNA amplicon sequences. For sequence classification, taxonomy was assigned using the in-house reference database curated by RTL Genomics. Prior to phylogenetic tree estimation, a multiple sequence alignment was created using MUSCLE v2.0 (doi.org/10.1093/nar/gkh340) and subsequent tree estimation was performed using FastTree2 (doi.org/10.1371/journal.pone.0009490), a software developed for interpreting large sequence alignments based on maximum likelihood. A phylogenetic tree was then constructed in the Newick format from a multiple sequence alignment of OTUs using MUSCLE [32, 33] and created in FastTree [34–36].

### Statistical analysis

Statistical analyses were conducted in R version 3.5.2 [37]. First, alpha diversity was expressed as the number of OTUs and as Shannon diversity. Case characteristics (i.e., PMI, age, sex) were evaluated for their influence on alpha diversity first using ANOVA. For beta diversity, compositional differences among samples were summarized using weighted UniFrac via the phyloseq package [38]. Multivariate differences among groups were evaluated with permutational multivariate analysis of variance (PERMANOVA) [39], using the R function ADONIS [39]. Backward feature selection was used to eliminate descriptors which were not significantly associated in ANOVA and PERMANOVA testing. Principal coordinates analysis (PCoA) using weighted UniFrac distances was performed. Mantel tests were carried out by conducting spearman correlations between a pairwise matrix of drug presence/absence and the microbial weighted UniFrac distance matrix. To evaluate differences in the relative abundances of taxa between cohorts, analysis of composition of microbiota (ANCOM) [40] was carried out on taxa present in at least 10% of samples and using moderate correction (multcorr = 2).

## Results

Prior to analysis, rarefaction survey was used to determine that samples were sequenced to sufficient depth to characterize microbial communities (S1 Fig in S1 File) with a median post-QC depth of 25,322 reads (1^st^ Q = 22,807, 3^rd^ Q = 32,061). In total, 36 cases passed QC and 1097 OTUs were identified across 395 species. Cases were split evenly between SUD (n = 18) and non-SUD (n = 18), though cases were skewed towards males (n = 30 males) and SUD cases were significantly younger (p = 0.012, median = 42.5 years, 1^st^ Q. = 32.25, 3^rd^ Q. = 48.75) than non-SUD cases (median = 56 years, 1^st^ Q = 42.25, 3^rd^ Q. = 66.25).

### Bacterial profiles associated with SUDs

The most abundantly detected taxa were *Bacteroides*, *Escherichia coli*, *Clostridium*, *Blautia*, and *Faecalbacterium prausnitzii* (S2 Fig in S1 File). The effect of SUDs on alpha diversity was assessed using Shannon Diversity and OTU abundance while controlling for possible covariates. Using either measure, alpha diversity in SUD-related cases were not found to be significantly different from non-SUD cases (ANOVA, *p* > 0.05, S3 Fig in S1 File). However, male cases were found to have significantly more OTUs than females (F = 2.51; df = 1, 30; *p* = 0.040).

**Fig 1:**
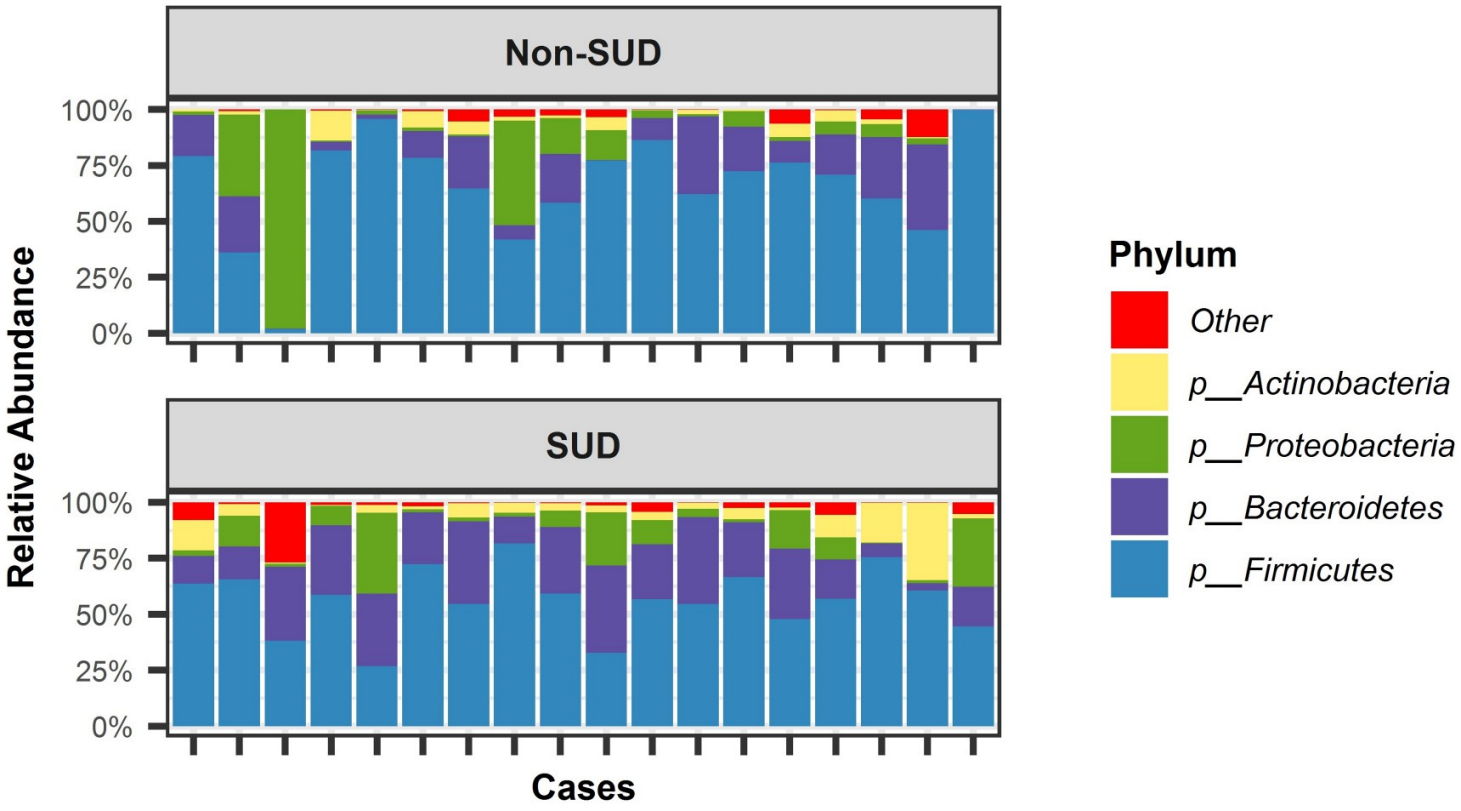
Depiction of four most prevalent bacteria detected per case and per SUD group. Welch’s non-parametric t-test shows that those groups are significantly different in their B:F population ratios.

Next, differences in overall composition (i.e., beta diversity) were investigated using a backward selected Permanova (ADONIS) model, where it was observed that SUD status was significantly associated to composition (F = 1.97; df = 1, 34; p = 0.024; R2 = 0.055, Fig 1A). No other covariates were found to significantly influence overall structure. Next, ANCOM procedure was used to determine if any species varied significantly by SUD group. Here, five species were identified to be differentially abundant, whereas *Eggerthella lenta* and *Blauti hansenii* were more abundantly distributed in SUD cases (Fig 1B).

**Fig 2:**
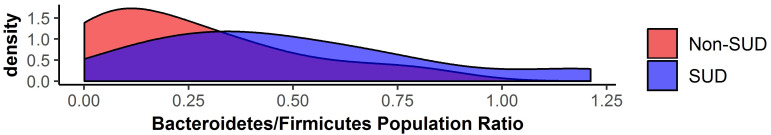
Density plot of the Bacteroidetes:Firmicutes (B:F) population ratio. Welch’s non-parametric t-test shows that those groups are significantly different in their B:F population ratios.

The overall distribution of phyla was also characterized between SUD and non-SUD samples, with *Firmicutes*, *Bacteroides*, *Proteobacteria* and *Actinobacteria* being the most abundant, in order (Fig 2). Notably, the Bacteroidetes/Firmicutes ratio (B:F ratio) was found to be significantly higher in SUD-related cases (mean = 0.49) than in non-SUD cases (mean = 0.26; Welch’s t-test: t = 2.36, adj. df = 30.8, p = 0.012, Fig 2), with a relative increase of 65% in SUD cases.

**Fig 3:**
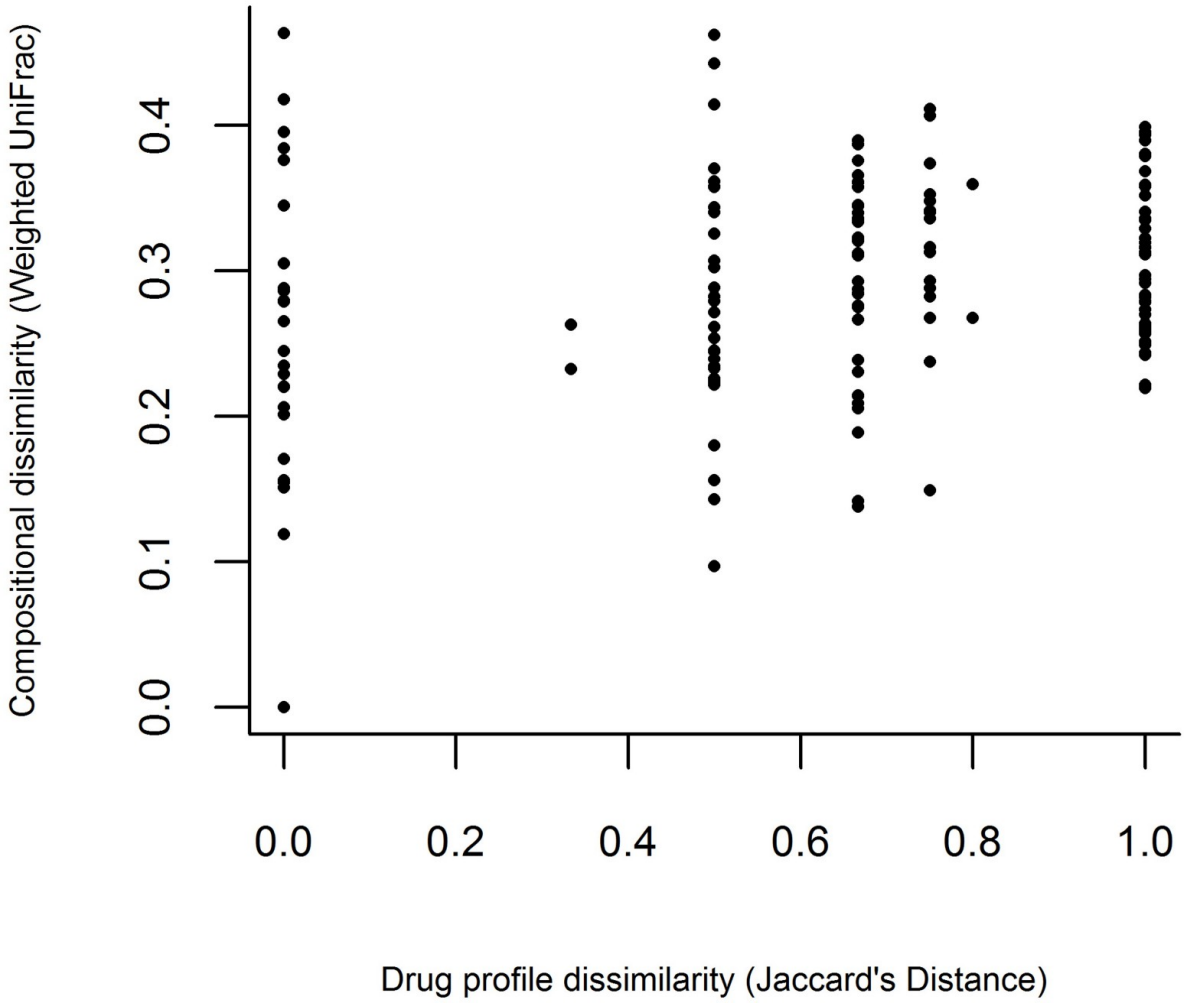
To test for differences in beta-diversity between groups the Jaccard distance metric was used. The unweighted Unifrac compositional dissimilarity was plotted against the Jaccard distances to demonstrate the drug profile dissimilarity.

**Fig 4:**
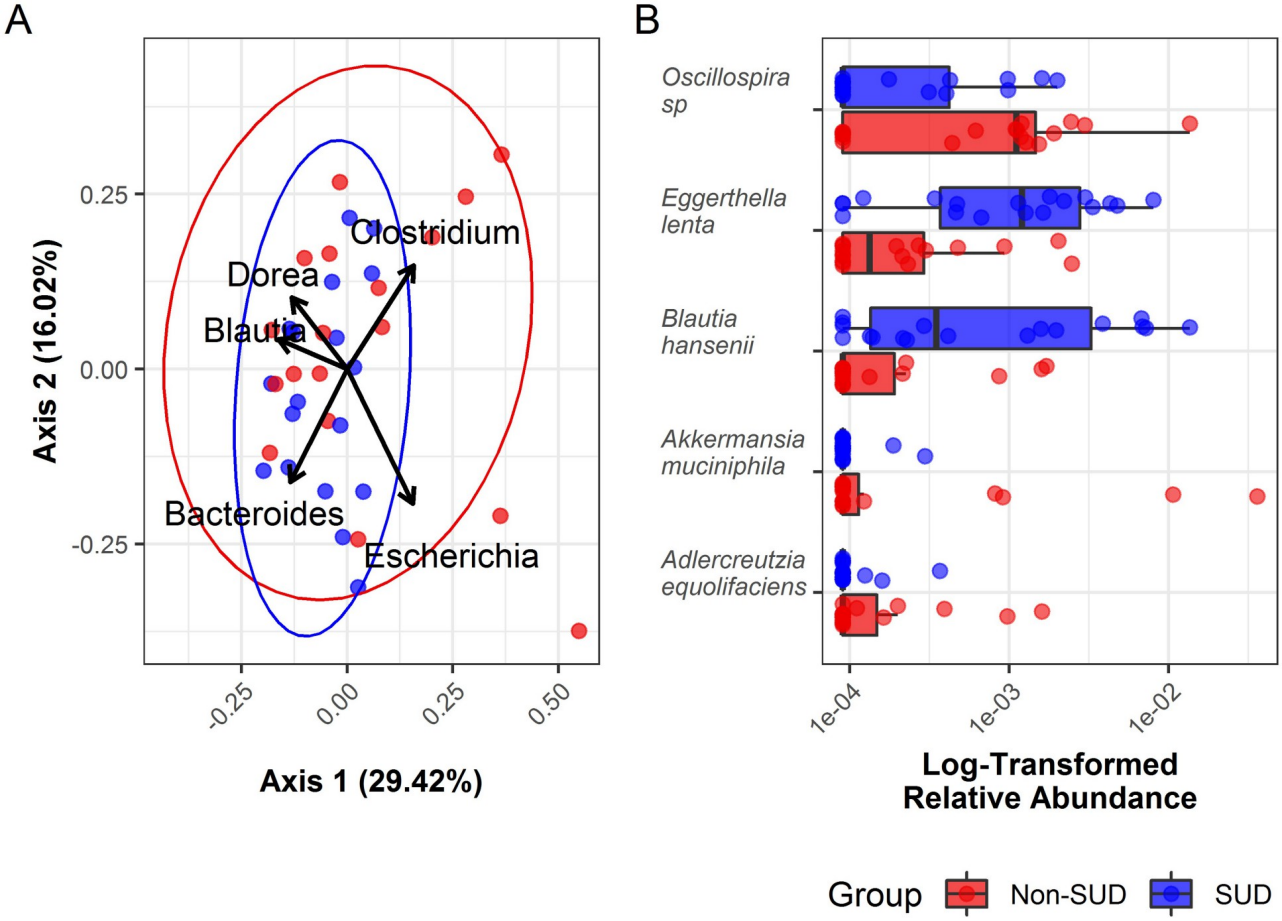
A. Projections of major microbial species contributing to the groups in the ordination plot. Biplot arrows indicate the top five genera abundances that accounted for the most variation in the ordination plot. B. The log-transformed relative abundances of bacterial taxa detected to be differentially abundant between groups by ANCOM are depicted by boxplot. The median value, first and third quartiles, and confidence interval in each group are illustrated.

Within SUD cases (n = 18), we next wanted to investigate whether specific drug profiles correlate with the gut microbiome. First, all cases substance matrix was established and summarized by Jaccard’s distance to be comparable to weighted UniFrac distance used to summarize microbial differences (Fig 3). A mantel test was then used to determine that the drug and microbiome profile distances were marginally correlated (Spearmans’s r = 0.25, p = 0.073, Fig 4), meaning cases where similar drugs were detected at autopsy also had slightly more similar overall microbial profiles.

## Discussion

The human gastrointestinal tract is home to an immense diversity of microbes (i.e., the gut microbiome), whereas disruption of this microbial community is linked to a plethora of disease states ranging from tumorigenesis and infection to, more recently, mood disorders and substance abuse [41, 42]. As most drug addiction studies of human postmortem neurological specimens focus on standard toxicological analyses of blood and urine, little is known regarding altered microbiome structure in severe cases, such as in individuals where substance abuse is a primary cause of death. Here, we presented a unique pathological examination of the colonic microbiome in postmortem cases which were categorized as deaths related or not related to SUDs.

The cadavers had PMIs ranging from one to 17 days. One of the limitations of this study relates to the time in which a dead body can have its autopsy performed. According to the Italian Regolamento di Polizia Mortuaria, Law number 285, Article 8 of 1990, autopsies are prohibited before 24 h after discovering a dead body [43]. Also in this study, PMIs and manners/causes of death were confirmed by official Daily Crime Logs. Another limitation is regarding the variables between cadavers. Unlike animal studies, the nature of this study negates the ability to control cadaver weight, age, PMI, drug types, cause of death, etc.

The notion that the gut microbiome and bidirectional signals impact mental health is not new, but in the past 15 years, this subject has garnered renewed awareness from researchers due to next generation sequencing technology, which provides a cost-effective, culture-independent method to study the structure and microbial diversity of complex communities. Most drug addiction studies of human postmortem neurological specimens focus on standard toxicological analyses of blood and urine. However, opioids generally tend to contain a heart blood concentration that is approximately half of the concentration found in the brain [44]. Therefore, we believe that postmortem brain tissue collected at autopsy is a better specimen than blood or urine and is more suited to provide a direct means to examine the neurobiology associated with SUDs. Furthermore, unlike animal tissue, whose circumstance at death can be controlled and manipulated, human tissue can only be collected naturalistically. Microorganisms found in internal organs are likely key players for understanding the health of large communities of people (e.g., drug overdose cases), and this could benefit the living [45].

As expected, microbial signatures that differentiate SUD cases from non-SUD were discovered (Figs 1 and 2), though with a small effect size as SUD status only explained 5% of variation among the two categories. The small effect size is not surprising, given the complexity of the gut microbiome it is reasonable that most lineages will be shared among postmortem tissues. However, the shift in overall community composition was further supported by enrichment of notable taxa. First, the overall mean abundance of Firmicutes was decreased in SUD cases (Fig 2). The B:F ratio was among the first notable features discovered in the gut microbiome and has been linked to gut barrier compromise and an infiltration of immune cells that leads to inflammation [18]. It is possible that a larger or more selective cohort will reveal microbial features with stronger associations [46]. Here, a non-significant (p = 0.073) but notable effect was observed where cases with similar drug profiles also had slightly more similar microbial composition (Fig 4). This observation, along with prior work documenting varied and substance-specific microbial responses [6], may hint at complex interactions that are dependent both on substance and functional pathway availability. Overall, the SUDs and non-SUDs groups had unequal B:F population ratios. The B:F population ratio was found to be significantly altered in SUD cases. This result supports Banerjee et al. (2016), who showed that opioids significantly alter gut microbiota, triggering preferential proliferation of Firmicutes and decreasing Bacteroidetes, effectively lowering population B:F ratios [18]. Based on these data, our working model is that further investigation into the composition of gut microbiota in SUDs cases is necessary to identify new diagnostics as well as therapeutic targets to prevent intentional and unintentional drug overdose, with the ultimate goal of restoring gut microbiome homeostasis.

Five species were found to be differentially enriched in SUD cases and the greatest disparity was observed in *Eggerthella lenta*, a lineage of well-known significance for its metabolic interactions with ingested drugs. First described in 1983, *E*. *lenta* (previously known as *Eubacterium lentum*) was isolated and found to actively convert the cardiac drug digoxin into a reduced form, dihydrodigoxin, in an arginine-dependent manner [47]. Since then, significant work has gone into characterizing the gene operon, now known as *cgr* (cardiac glycoside reductase), and its role in cardiac drug inactivation [48]. For example, it is now known that strains possessing the *cgr* operon were inhibited in their inactivation of digoxin by increased arginine levels. Though the present study did not have full access to the medical history of the included cases, the noted association between *E*. *lenta* abundance and SUD cases (Fig 1B) may suggest further uncharacterized pharmacological interactions. Indeed, a more recent study by Rekdal et al. (2019) reported a two-step inactivation of Levodopa (L-DOPA), used to treat Parkinson’s disease, involving *Enterococcus faecalis* and *E*. *lenta* using an apparently separate gene family [49]. In the scenarios involving digoxin and Levodopa, interest in characterizing these processes occurred because of undesirable drug inactivations, whereas such interactions between the gut microbiome and harmful substances such as opioids may be favorable depending on the nature of these reactions. Further research is necessary to elucidate the metabolic function and consequences of elevated *E*. *lenta* in SUD cases and in individuals affected by substance abuse.

Across a large spectrum of health and disease, the gut microbiome is increasingly being considered as a therapeutic target, whether by diet modification, probiotics, or even via transplantation of whole communities (e.g., fecal matter transplant) [50]. If such an approach is to be adapted to mood and SUDs, a comprehensive understanding of the associated microbiome communities and their altered functions will be necessary. Our findings implicate *E*. *lenta* in a currently unknown role related to SUDs, plausibly through interaction with substance-related metabolism as in previous reports [51]. We have not considered at this time whether *E*. *lenta* is performing a helpful or harmful function, as previous reports may suggest that substance inactivation reduces gastrointestinal toxicity from those substances [52] or, as in the case of Levodopa, metabolic processes resulting in increased dopamine levels may endorse addictive behaviors [49]. The current study furthers emerging literature of postmortem microbial communities, which will ultimately contribute to advanced knowledge of the effects of drug use on putrefaction of human corpses. Overall, we expect that continued studies into the altered state of the microbiome in addiction and substance use will reveal novel targets for therapy.

## Supporting information

S1 File Consists of ALL the supplementary figures and tables for this submission. (PDF)
