# Correction: Correlation between postmortem microbial signatures and substance abuse disorders

**DOI:** 10.1371/journal.pone.0319427

**Published:** 2025-02-12

**Authors:** Gulnaz T. Javan, Tiara Wells, Jamese Allen, Silvia Visona, Matteo Moretti, Craig Tipton, Latia Scott, Sheree J. Finley

In the Funding section, the grant number from the funder National Science Foundation is listed incorrectly. The correct grant number is: HRD 2011764 and HRD 2151000.
