# Supplementary material for: Correlation between postmortem microbial signatures and substance abuse disorders
Source: PLoS One. 2022 Sep 26;17(9):e0274401. doi: 10.1371/journal.pone.0274401 (PMC9512198; doi:10.1371/journal.pone.0274401)
Supplement: S1 File — (PDF) [file pone.0274401.s001.pdf]

Table S1. Demographic data were collected on each of the 40 corpses: ethnicity, height, weight, PMI, cause of death, manner of death, SUDs status, and drug substance.

Case Number	Ethnicity	Height (cm)	Weight (kg)	PMI (days)	Cause of Death	Manner of Death	Non-Drug Related	Drug Addicted	Substance
Case-1	Caucasian	160	65	5 days	Thoracic Trauma	Accident	X		
Case-2	Caucasian	176	65	7 days	Sepsis	Natural Death	X		
Case-3	Caucasian	160	47	3 days	Cardiac Rupture	Natural Death	X		
Case-4	Caucasian	176	90	6 days	Drug Overdose	Accident		X	Methadone-ketamine
Case-5	Caucasian	163	55	3 Days	Acute Cocaine Intoxication	Accident		X	Cocaine/methadone
Case-6	Caucasian	160	70	4 Days	Drug Overdose	Accident		X	Cocaine-morphine
Case-7	Caucasian	178	80	1 Day	Ischemic Heart Disease	Accident	X		
Case-8	Caucasian	177	75	7 Days	Hemorrhagic Shock	Accident		X	Cocaine
Case-9	Caucasian	177	95	4 Days	Acute Cardiac Failure	Natural Death	X		
Case-10	Caucasian	177	80	8 Days	Drug Overdose	Accident		X	Cocaine
Case-11	Caucasian	167	85	2 Days	Drug Overdose	Accident		X	Cocaine-morphine
Case-12	Caucasian	181	100	5 Days	Cardiac Failure	Natural Death	X		
Case-13	Caucasian	174	75	5 Days	Acute Myocardial Ischemia	Accident	X		
Case-14	Caucasian	180	70	2 Days	Drug Overdose	Accident		X	Heroin
Case-15	Caucasian	173	80	7 Days	Cardiac Failure	Natural Death	X		
Case-16	African American	180	60	10 Days	Drowning	Accident		X	Alcohol
Case-17	Caucasian	175	59	1 Day	Esophageal Varices Bleeding	Natural Death	X		
Case-18	Caucasian	147	60	5 Days	Respiratory Failure	Accident	X		
Case-19	Caucasian	172	70	3 Days	Drug Overdose	Accident		X	Heroine/cocaine
Case-20	Caucasian	180	56	4 Days	Drug Overdose	Accident		X	Methadone-cocaine-benzadiazepines
Case-21	Caucasian	172	80	3 Days	Acute Myocardial Ischemia	Accident	X		
Case-22	Caucasian	180	75	6 Days	Acute Myocardial Infarction	Natural Death	X		
Case-23	Caucasian	169	68	7 Days	Hemorrhagic Shock	Accident		X	Cocaine-morphine
Case-24	Caucasian	179	95	3 Days	Acute Cocaine Intoxication	Accident		X	Cocaine
Case-25	Caucasian	179	65	2 Days	Asphyxia (Hanging)	Suicide		X	Cocaine
Case-26	Caucasian	161	68	3 Days	Sudden Cardiac Death	Accident	X		
Case-27	Caucasian	183	75	4 Days	Drug Overdose	Accident		X	Cocaine
Case-28	Caucasian	174	60	2 Days	Acute Heroine Intoxication	Accident		X	Cocaine
Case-29	Caucasian	164	60	5 Days	Acute Cardiac Failure	Natural Death		X	Unknown/alcohol
Case-30	Caucasian	173	80	7 Days	Cardiac Failure	Natural Death	X		
Case-31	Caucasian	168	68	2 Days	Acute Cardiac Insufficiency	Natural Death	X		
Case-32	Caucasian	179	70	9 Days	Drug Overdose	Natural Death	X		
Case-33	Caucasian	180	72	3 Days	Drug Overdose	Accident		X	Methadone-cocaine-benzadiazepines
Case-34	Caucasian	175	64	3 Days	Head Trauma	Accident	X		
Case-35	Caucasian	176	140	2 Days	Drug Overdose	Accident		X	Cocaine, heroine, oxycodone
Case-36	Caucasian	177	67	5 Days	Drug Overdose	Accident		X	
Case-37	Caucasian	181	80	17 Days	Hemorrhagic Shock	Accident	X		
Case-38	Caucasian	172	80	5 Days	Pulmonary Thromboembolism	Natural Death	X		
Case-39	Caucasian	149	40	4 Days	Bilateral Pneumonia	Natural Death	X		
Case-40	Caucasian	170	65	10 Days	Drug Overdose	Accident		X	Heroine, cocaine, benzodiazepamine

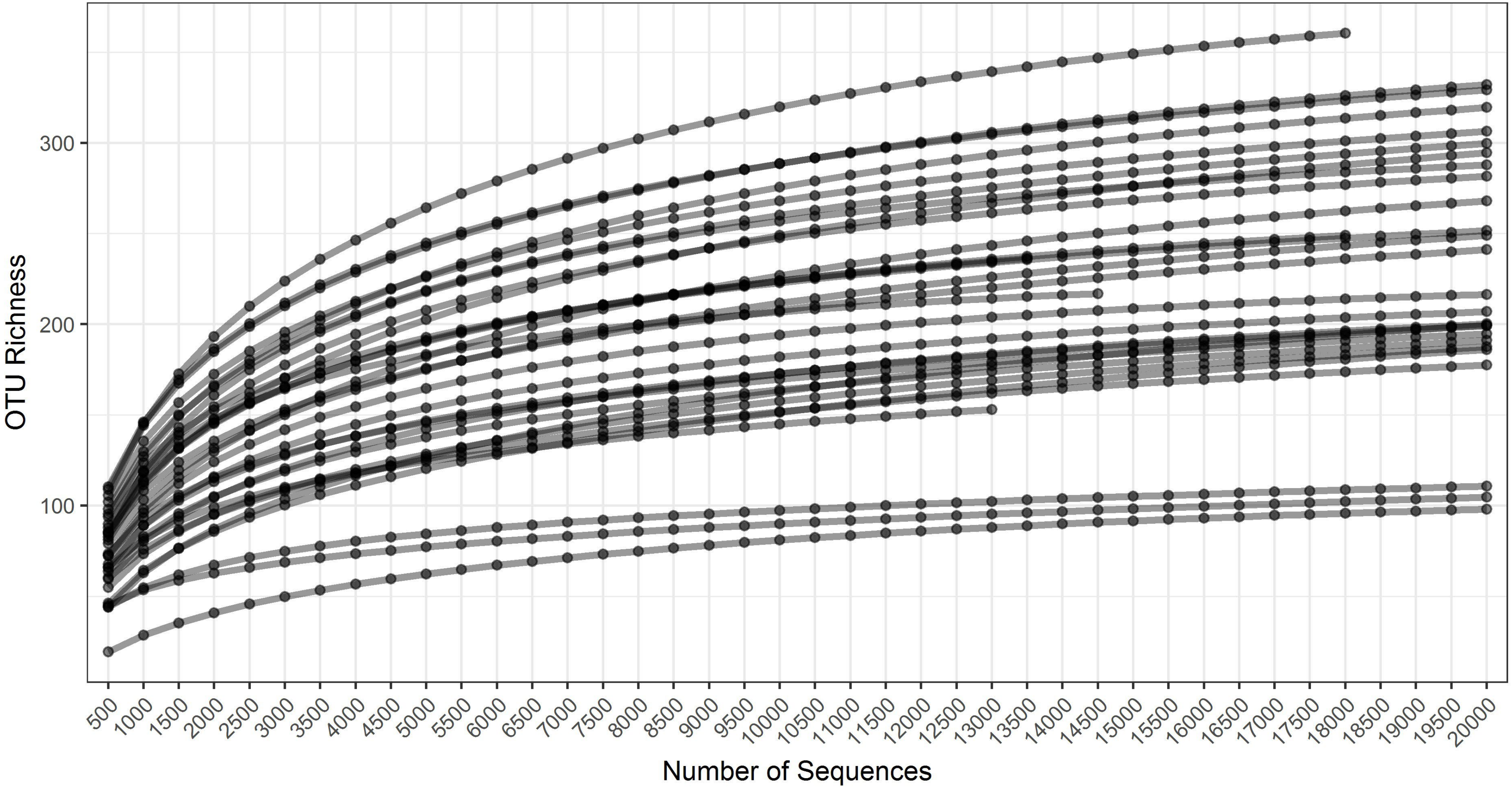

S1 Fig. Rarefaction survey to determine the sequencing depth to a minimum depth of 10,000 reads required to characterize microbial communities. Each curve reached asymptote indicative of adequate reads for comparison of the microbiomes.

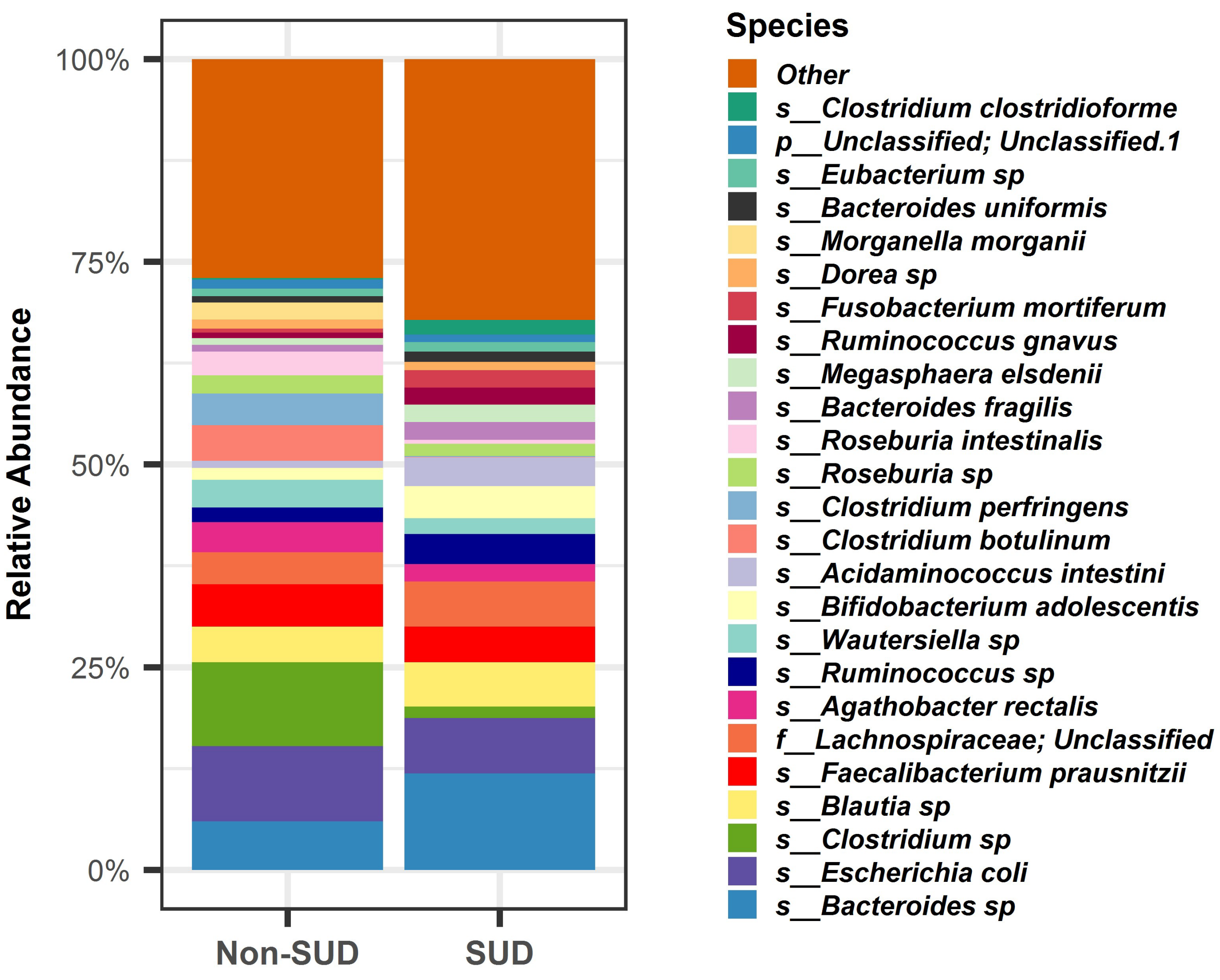

S2 Fig. Microbiome profile of the samples for SUD and non-SUD cases. Shown are the 25 most predominant species based on the relative abundance of per group.

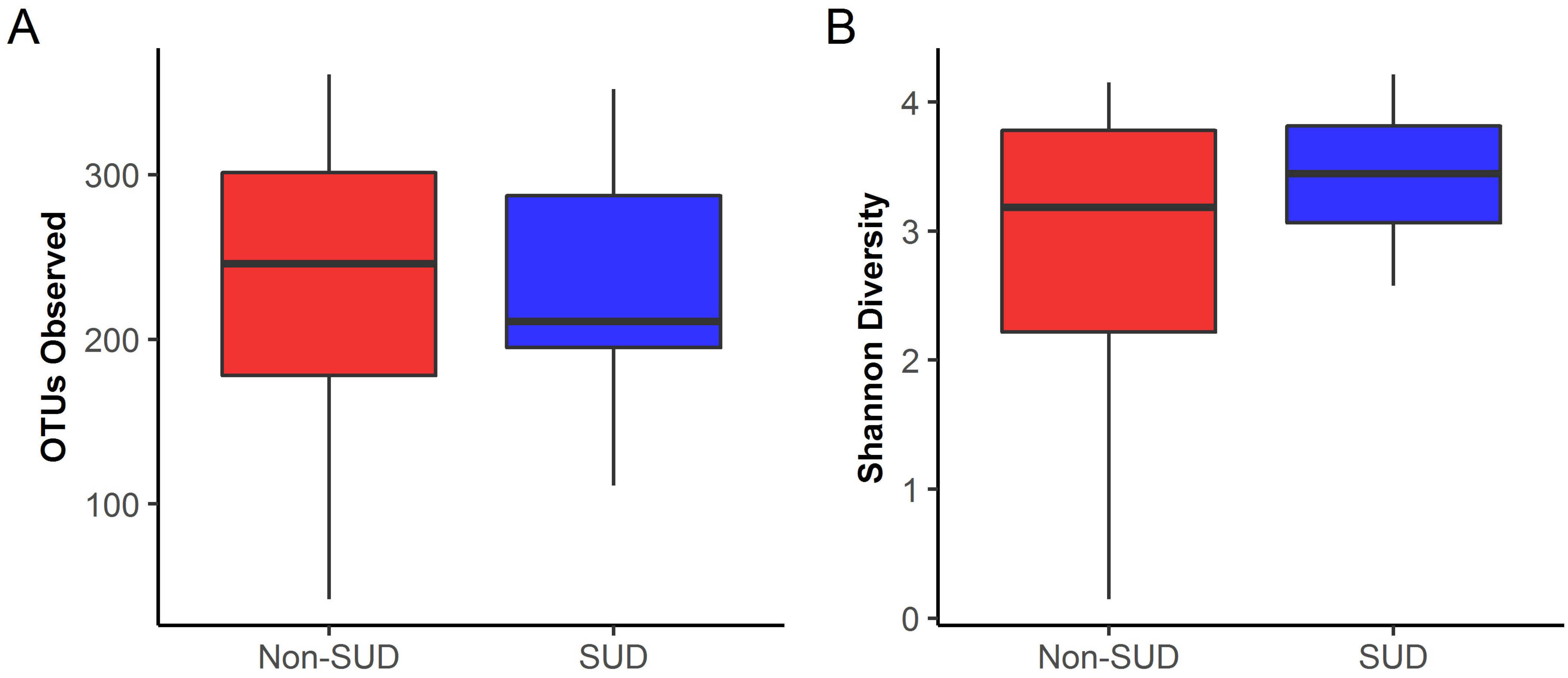

S3 Fig. Boxplots of alpha diversity depicting the relationship of A) non-SUD and SUD cases with OTU richness and B) non-SUD and SUD cases with Shannon Diversity.
